# Role of miRNA‐542‐5p in the tumorigenesis of osteosarcoma

**DOI:** 10.1002/2211-5463.12824

**Published:** 2020-03-18

**Authors:** Tengjiao Zhu, Daoyang Fan, Kaifeng Ye, Bingchuan Liu, Zhiyong Cui, Zhongjun Liu, Yun Tian

**Affiliations:** ^1^ Department of Orthopedic Third Hospital of Peking University Beijing China

**Keywords:** bioinformatics, biomarker, miRNA, miRNA‐542‐5p, osteosarcoma

## Abstract

Osteosarcoma, one of the most common malignant bone tumors, is characterized by a high rate of metastasis, and the survival rate of patients with metastatic osteosarcoma is poor. Previous studies have reported that miRNAs often regulate the occurrence and development of various tumors. In this work, we identified miRNA‐542‐5p as a critical miRNA in osteosarcoma by overlapping three Gene Expression Omnibus datasets, and then evaluated miRNA‐542‐5p expression profiles using Gene Expression Omnibus and Sarcoma‐microRNA Expression Database. We used MISIM to investigate miRNAs correlated with miR‐542 and identified potential target genes of miRNA‐542‐5p using miRWalk. Functional and pathway enrichment analyses were performed using The Database for Annotation, Visualization and Integrated Discovery. Protein–protein interaction was performed using Search Tool for the Retrieval of Interacting Genes and Cytoscape. We report that the relative level of miRNA‐542‐5p was significantly higher in osteosarcoma than in healthy bone. Expressions of hsa‐miR‐330 and hsa‐miR‐1202 were found to be strongly correlated with that of miR‐542‐5p. Furthermore, we identified a total of 514 down‐regulated genes as possible targets of miR‐542‐5p. Gene Ontology and Kyoto Encyclopedia of Genes and Genomes analysis demonstrated that the putative target genes of miR‐542‐5p were most enriched in the cell‐cycle process. The differentially expressed genes *CDCA5*, *PARP12* and HSPD1 were found to be hub genes in protein–protein interaction networks. Finally, transfection of the osteosarcoma cell line U2OS with miR‐542‐5p mimics or inhibitor revealed that miR‐542‐5p can promote cell proliferation. In conclusion, our results suggest that miR‐542‐5p may promote osteosarcoma proliferation; thus, this miRNA may have potential as a biomarker for diagnosis and prognosis.

## Graphical abstract

Osteosarcoma is characterized by a high metastasis rate and low survival rate. In this article, we observed that the expression of miR‐542‐5p was significantly increased in osteosarcoma compared with normal bones, and miR‐542‐5p promoted proliferation in osteosarcoma cells. Therefore, miR‐542‐5p may be a promising biomarker in the early diagnosis of patients with osteosarcoma.

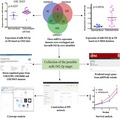

**Abbreviations**
BP: biological process
CCK‐8: Cell Counting Kit‐8
DAVID: Database for Annotation, Visualization and Integrated Discovery
FC: fold change
GEO: Gene Expression Omnibus
GO: Gene Ontology
KEGG: Kyoto Encyclopedia of Genes and Genomes
miRNA: microRNA
PPI: protein–protein interaction
STRING: Search Tool for the Retrieval of Interacting Genes
S‐MED: Sarcoma‐microRNA Expression Database
TCGA: The Cancer Genome Atlas

Osteosarcoma, one of the most common malignant bone tumors, is characterized by a high rate of metastasis [1]. Eighty percent of patients have been associated with lung metastases at the time of disease diagnosis [2]. Benefitting from the improvement of surgical techniques and the development of neoadjuvant chemotherapy, the classic high‐grade osteosarcoma prognosis has significantly improved from <20% survival to up to 70–80% in patients without clinically obvious metastatic disease [3]. However, the overall survival rate of patients with metastatic osteosarcoma is still not optimistic [4]. Therefore, it is urgent to find a biomarker for early diagnosis in osteosarcoma.

MicroRNA (miRNA) is a group of short single‐stranded noncoding RNAs (about 21–24 nucleotides long) and plays an important role in regulating gene expression by binding to the 3′ UTR of mRNAs [5]. Previous studies have demonstrated that miRNAs are widely involved in various cellular biological processes (BPs), such as cell proliferation [6], apoptosis [7] and cell cycle [8] in osteosarcoma. Therefore, the differential expression levels of miRNA indicate their potential role as diagnostic biomarkers in osteosarcoma.

In our study, data from the Gene Expression Omnibus (GEO) database and The Cancer Genome Atlas (TCGA) database demonstrated that miR‐542‐5p are significantly up‐regulated in osteosarcoma tissues or cell lines compared with the normal bones. Currently, few studies are integrating microarray datasets to investigate key miRNAs in osteosarcoma. Hence the objective of this work aimed to evaluate whether miR‐542‐5p could be a novel biomarker for the early diagnosis of osteosarcoma.

## Materials and methods

### GEO database

GEO (http://www.ncbi.nlm.nih.gov/geo) is a public functional genomics data repository for high‐throughput gene expression data, different types of microarrays and chips [9]. The miRNA microarray datasets (http://www.ncbi.nlm.nih.gov/geo/query/acc.cgi?acc=GSE69470 [10], http://www.ncbi.nlm.nih.gov/geo/query/acc.cgi?acc=GSE28425 [11] and http://www.ncbi.nlm.nih.gov/geo/query/acc.cgi?acc=GSE65071 [12]) were downloaded from GEO (GPL20275, GPL8227 and GPL19631 platform, respectively), and we performed an integrated analysis. Comprehensive miRNA expression profiling was obtained by using the probes in the platforms mentioned earlier. The http://www.ncbi.nlm.nih.gov/geo/query/acc.cgi?acc=GSE69470 dataset contained 10 human osteosarcoma cell lines and 5 normal cell lines. The http://www.ncbi.nlm.nih.gov/geo/query/acc.cgi?acc=GSE28425 dataset contained 19 human osteosarcoma cell lines and 4 human normal bones. The http://www.ncbi.nlm.nih.gov/geo/query/acc.cgi?acc=GSE65071 dataset contained 20 plasma samples from osteosarcoma patients and 15 healthy controls. Moreover, we further investigated the differential expression of miR‐542‐5p in human osteosarcoma cell lines and human normal bones by analyzing the http://www.ncbi.nlm.nih.gov/geo/query/acc.cgi?acc=GSE28425 dataset.

Furthermore, we acquired a list of down‐regulated genes by analyzing three gene expression datasets: http://www.ncbi.nlm.nih.gov/geo/query/acc.cgi?acc=GSE32395, [13] GSE36004 [14] and http://www.ncbi.nlm.nih.gov/geo/query/acc.cgi?acc=GSE70415 [15]. *P* < 0.05 and log_2_FC < −1 were used to screen statistically significant genes. The http://www.ncbi.nlm.nih.gov/geo/query/acc.cgi?acc=GSE32395 dataset contained seven human osteosarcoma cell lines and two normal cell lines. The http://www.ncbi.nlm.nih.gov/geo/query/acc.cgi?acc=GSE36004 dataset contained 19 human osteosarcoma cell lines, 4 human normal bones and 2 osteoblasts. The http://www.ncbi.nlm.nih.gov/geo/query/acc.cgi?acc=GSE70415 dataset contained five human osteosarcoma cell lines and one normal cell line.

### Tumor‐miRNA‐Pathway database

Tumor‐miRNA‐Pathway database is a newly developed online database by Guo lab (http://bioinfo.life.hust.edu.cn/miR_path/index.html). As an interactive website, Tumor‐miRNA‐Pathway provides the miRNA pathway regulation and their expressions in the various cancer types. In this study, we obtained the miR‐542‐5p and miR‐874‐3p expression profile of different tumor types and adjacent normal samples from the TCGA data analysis tool.

### MISIM v2.0 database

MISIM v2.0 (http://www.lirmed.com/misim) is open accessed and based on miRNA‐disease data from the HMDD v3.0 database, and the miRNA–disease data were curated using miRBase (Release 22.1). Currently, MISIM v2.0 contains 14 250 miRNA–disease associations, including 1044 miRNAs and 613 diseases, and 547 miRNA sets from TAM 2.0. MISIM v2.0 can offer the miRNA functional similarity in more detail (e.g., stratifying by up‐ and down‐miRNAs), higher quality and more accurate enrichment analysis to users. Moreover, MISIM v2.0 also can predict the novel association diseases for the interested miRNAs [16]. MISIM v2.0 was used to analyze the correlated miRNAs with hsa‐miR‐542 and hsa‐miR‐874 in cancers.

### Sarcoma‐microRNA expression database

Sarcoma‐microRNA Expression Database (S‐MED) is a repository that describes the patterns of miRNA expression found in various human sarcoma tumor types and select normal tissues. The database also provides statistical details, such as fold changes (FCs) and *P*‐values for differentially expressed miRNAs in each sarcoma and normal tissue type [17]. In this work, we explored miR‐542‐5 expression in different types of sarcoma and normal bones.

### miRWalk database

miRWalk (http://mirwalk.umm.uni-heidelberg.de) is a freely accessible platform that provides the miRNA target site prediction [18]. Moreover, it integrates convincing miRNA target predicted results from other databases, including Microt4, miRanda, mirBridge, miRDB, miRmap, miRNAMap, Pictar2, PITA, RNA22, RNA hybrid and TargetScan. We collected the possible miR‐542‐5p target genes with this database for further analysis. To improve the accuracy of the results, we considered the down‐regulated genes both in the three GEO datasets mentioned earlier and the predicted miRNA target in miRWalk. The overlapping genes were used for conducting biometric analysis to explore potential molecular mechanisms of miR‐542‐5p in osteosarcoma.

### Database for Annotation, Visualization and Integrated Discovery

The Database for Annotation, Visualization and Integrated Discovery (DAVID; https://david.ncifcrf.gov/home.jsp) is a web‐accessible program and provides a comprehensive set of functional annotation tools for investigators to understand biological meaning behind large lists of genes [19]. We uploaded the overlapping gene list into the database and enriched a list of Gene Ontology (GO) and Kyoto Encyclopedia of Genes and Genomes (KEGG) pathways by functional analysis based on the standard false discovery rate < 0.05 and *P* < 0.05.

### Search Tool for the Retrieval of Interacting Genes database

Search Tool for the Retrieval of Interacting Genes (STRING; https://string-db.org) is an online website to predict protein–protein interactions (PPIs) [20]. Each node and line in the network represent the target gene and interaction, respectively. We investigated the interactions between the target genes of miR‐542‐5p and constructed the PPI networks using the STRING database.

### Cytoscape database and related plugins

Cytoscape (version 3.4.2) is an open bioinformatics platform for constructing protein interaction networks [21]. We imported the PPI network data from the STRING database into Cytoscape and identified the most densely connected regions and module analysis by using the Molecular Complex Detection plugin [22]. Besides, ClueGO is a Cytoscape plugin to integrate GO terms, as well as KEGG pathways, and create functionally organized networks [23]. CluePedia Cytoscape plugin [24] is a user‐friendly tool for visualizing genes associated to related pathways. In this study, ClueGO plus CluePedia plugins were used to interpret the links between the significant genes and related pathways in enriched GO terms and KEGG terms in the osteosarcoma.

### Cell line and cell culture

The osteosarcoma cell line U2OS (bought from China Infrastructure of Cell Line Resources, Beijing, China) was selected for the following experiments. The cells were cultured in McCoy’s 5A medium (Gibco, Grand Island, NY, USA) supplemented with 10% FBS (Gibco), 100 U·mL^−1^ penicillin and 100 μg·mL^−1^ streptomycin. The cells were maintained at 37 °C in a humidified atmosphere containing 5% CO_2_.

### Transfection

For exploring gain‐ and loss‐of‐function studies of miR‐542‐5p, U2OS cells were transfected by miRNA mimics and inhibitors (synthesized by JiMa) using liposomal transfection reagent (bought from YeSen) for 2 consecutive days. The sequences were as follows: miR‐542‐5p‐mimic (5′‐TCGGGGATCATCATGTCACGAGA‐3′); miR‐542‐5p‐inhibitor (5′‐TCTCGTGACATGATGATCCCCGA‐3′). The cells were harvested 24 h after the last transfection for the next experiments.

### Cell proliferation assay

After 2 consecutive days of transfection, 4000 U2OS cells were seeded into each well of a 96‐well plate and incubated for further analysis. The proliferation was determined by using Cell Counting Kit‐8 (CCK‐8; Dojindo, Kumamoto, Kyushu, Japan), and the absorbance was measured at 450 nm. The results were performed at least three times independently.

### Statistical analysis

The results were shown as the mean ± standard deviations (SDs) and analyzed with Student’s *t*‐test. *P* < 0.05 was considered statistically significant.

## Results

### Identification of differentially expressed miRNAs

**Fig. 1:**
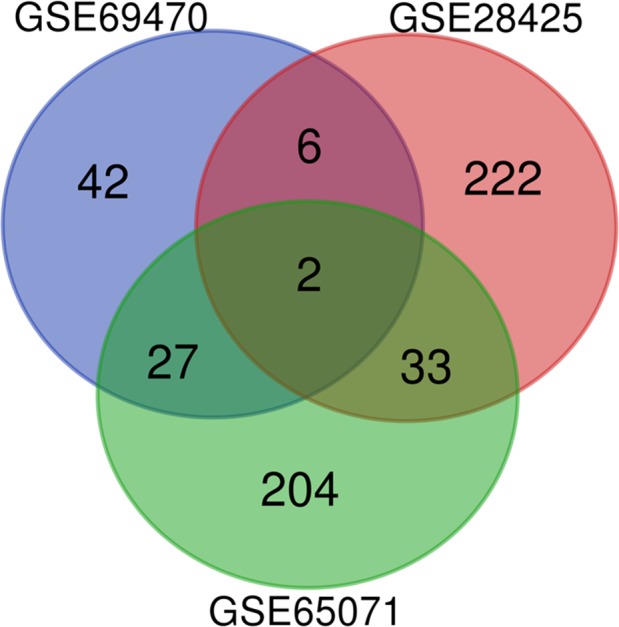
Venn diagram. The three miRNA expression datasets were overlapped, and two miRNAs (hsa‐miR‐542‐5p and hsa‐miR‐874) were identified as the key miRNAs in osteosarcoma.

After analysis of the microarray results, there were 77 differentially expressed miRNAs (*P* < 0.05) between osteosarcoma and normal cells in http://www.ncbi.nlm.nih.gov/geo/query/acc.cgi?acc=GSE69470. In http://www.ncbi.nlm.nih.gov/geo/query/acc.cgi?acc=GSE28425, 263 miRNAs were identified as differentially expressed miRNAs (*P* < 0.05). Besides, 266 miRNAs were substituted in http://www.ncbi.nlm.nih.gov/geo/query/acc.cgi?acc=GSE65071. The overlap among the three datasets was shown in the Venn diagram (Fig. 1), and two miRNAs (hsa‐miR‐542‐5p and hsa‐miR‐874) were obtained (Data S1). We selected hsa‐miR‐542‐5p for further study.

### The clinical significance of miR‐542‐5p from TCGA

**Fig. 2:**
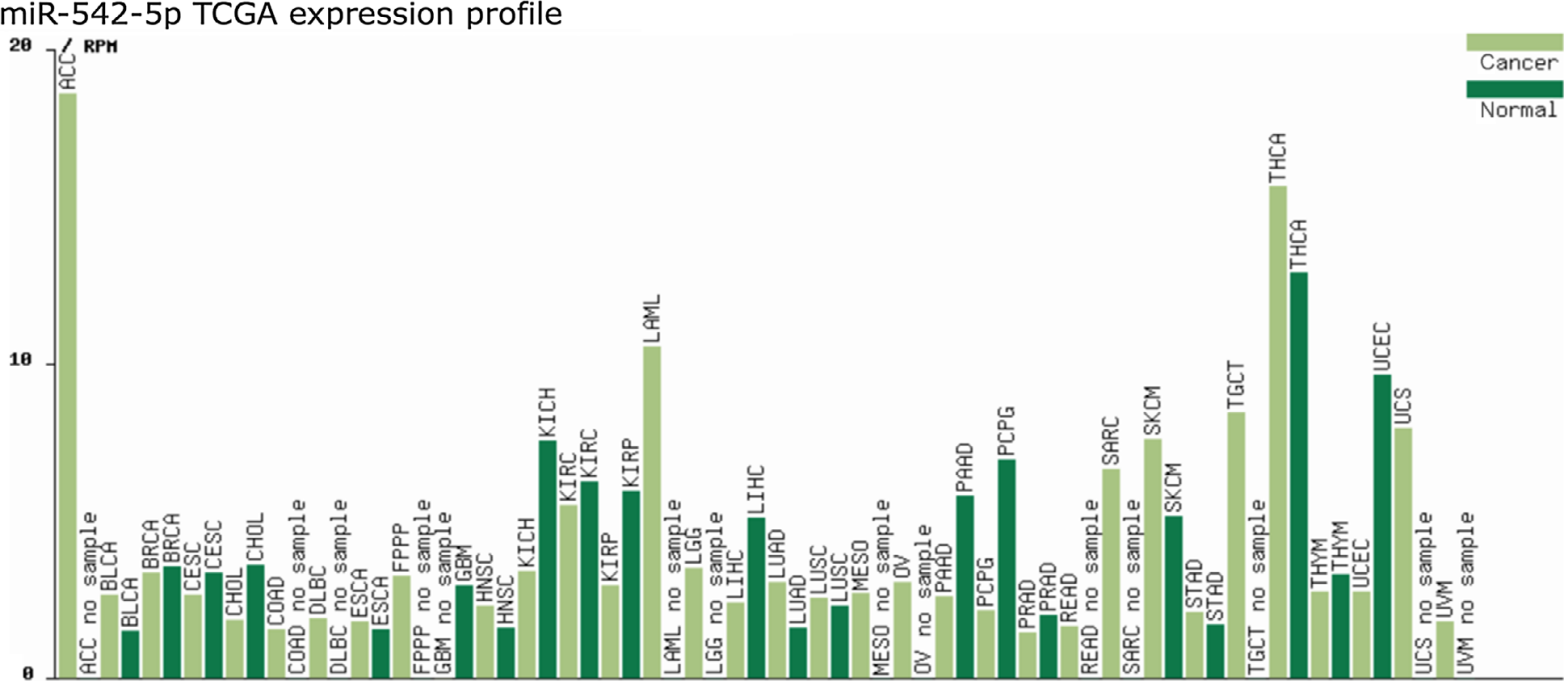
Expression profile of miR‐542‐5p in different cancer types from TCGA.

As shown in Fig. 2, the expression profiling of miR‐542‐5p indicated that it was underexpressed in various human cancer types, such as kidney cancer, liver cancer and pancreatic adenocarcinoma. Therefore, miR‐542‐5p might act as tumor suppressor gene. However, miR‐542‐5p could promote tumorigenesis in several cancers, including head and neck squamous cell carcinoma, skin cutaneous melanoma and thyroid carcinoma, of which miR‐542‐5p was found to be higher in the cancer samples than that in the normal one. Unfortunately, the expression level of miR‐542‐5p in osteosarcoma was not provided by TCGA database.

### Coexpressed miRNA with miR‐542‐5p

**Fig. 3:**
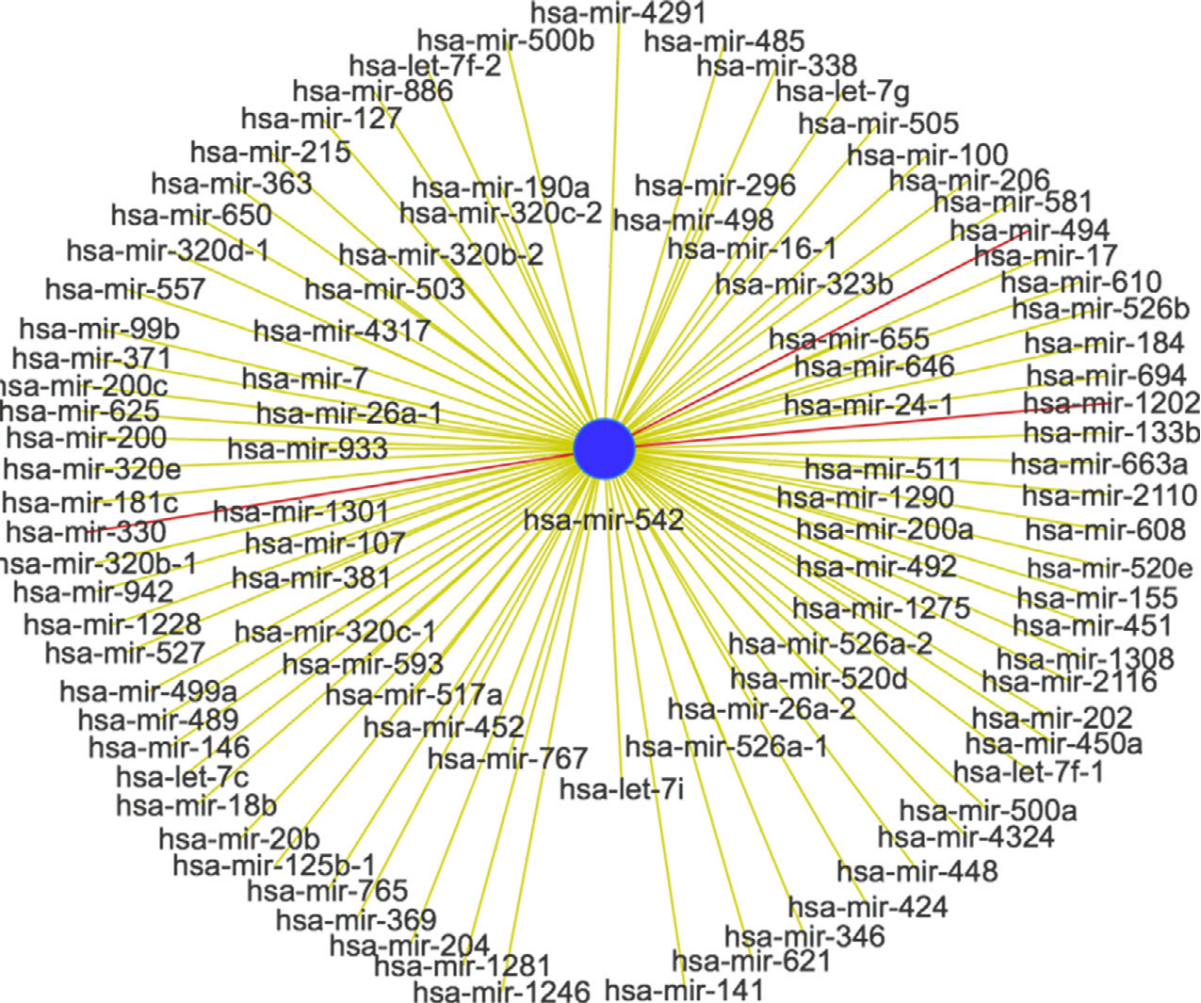
Diagram of correlated miRNAs with hsa‐miR‐542.

The coexpressed miRNAs with miR‐542‐5p were analyzed by the MISIM database, and the correlated miRNAs with miR‐542‐5p were shown in Fig. 3. Besides, hsa‐miR‐330, hsa‐miR‐494 and hsa‐miR‐1202 were the most connected miRNAs with miR‐542‐5p (threshold value: 0.5–1) according to analysis results.

### miR‐542‐5p was overexpressed in osteosarcoma

**Fig. 4:**
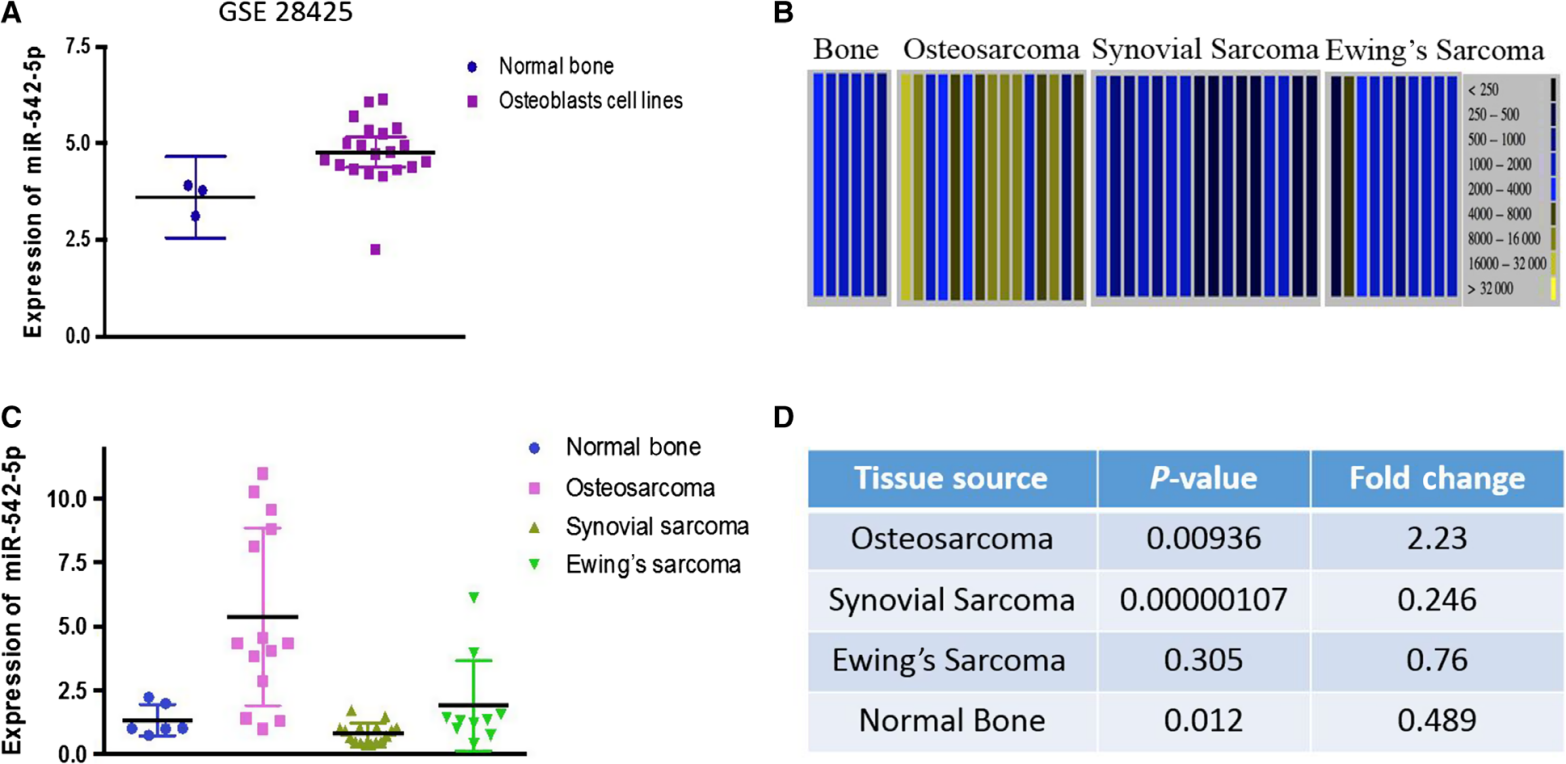
Expression of miR‐542‐5p in osteosarcoma based on GEO and S‐MED databases. (A) http://www.ncbi.nlm.nih.gov/geo/query/acc.cgi?acc=GSE28425 (*P* = 0.0297). (B) Absolute intensity data view in S‐MED database; the *y* axis represents microarray intensity. (C) Diagram of numeric data. (D) *P*‐value and FC of four sarcomas.

We investigated the expression difference of osteosarcoma cell lines by analyzing 19 human osteosarcoma cell lines and three human normal bones in the http://www.ncbi.nlm.nih.gov/geo/query/acc.cgi?acc=GSE28425 dataset. As shown in Fig. 4A, the expression of miR‐542‐5p in osteosarcoma cell lines was significantly overexpressed compared with that of normal bones (*P* = 0.0297). The mean ± standard deviation of osteosarcoma cell lines and normal bones were 4.769 ± 0.1872 and 3.603 ± 0.2452, respectively. Furthermore, we explored the differential expression of miR‐542‐5p in patients with osteosarcoma and adjacent normal tissues based on the S‐MED database. The database provided expression data for miR‐542‐5p in four bone‐associated sarcomas (Fig. 4B), including osteosarcoma (*n* = 14), synovial sarcoma (*n* = 16), Ewing’s sarcoma (*n* = 10) and normal bone (*n* = 6). The analysis indicated that miR‐542‐5p was significantly up‐regulated in patients with osteosarcoma in comparison with normal bone and the other three bone‐related sarcomas (Fig. 4C,D). Therefore, the results mentioned earlier further demonstrated that miR‐542‐5p was overexpressed in osteosarcoma.

### Possible target genes of miR‐542‐5p

Possible target genes of miR‐542‐5p were identified by differential expression analysis based on three GEO datasets and the miRWalk database. Eventually, a total of 514 down‐regulated genes (*P* < 0.05 and log_2_FC < −1) were selected as possible target genes of miR‐542‐5p.

### GO and KEGG enrichment analyses of target genes

**Fig. 5:**
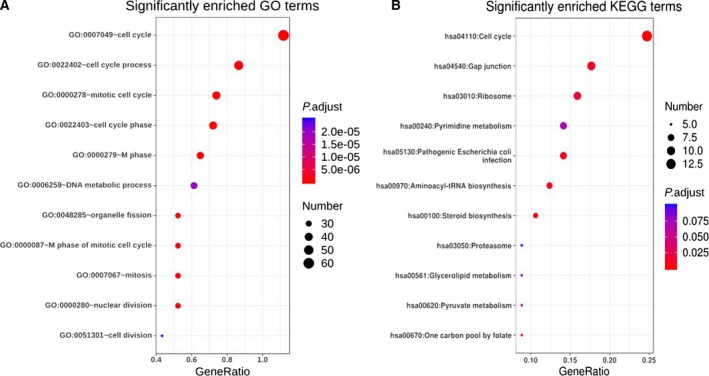
Overview of significant enriched GO and KEGG pathways. Each bubble indicates a term. The size of the bubble represents the number of involved genes. Lighter colors indicate smaller *P*‐values. (A) Enriched GO terms. (B) Enriched KEGG terms.

**Fig. 6:**
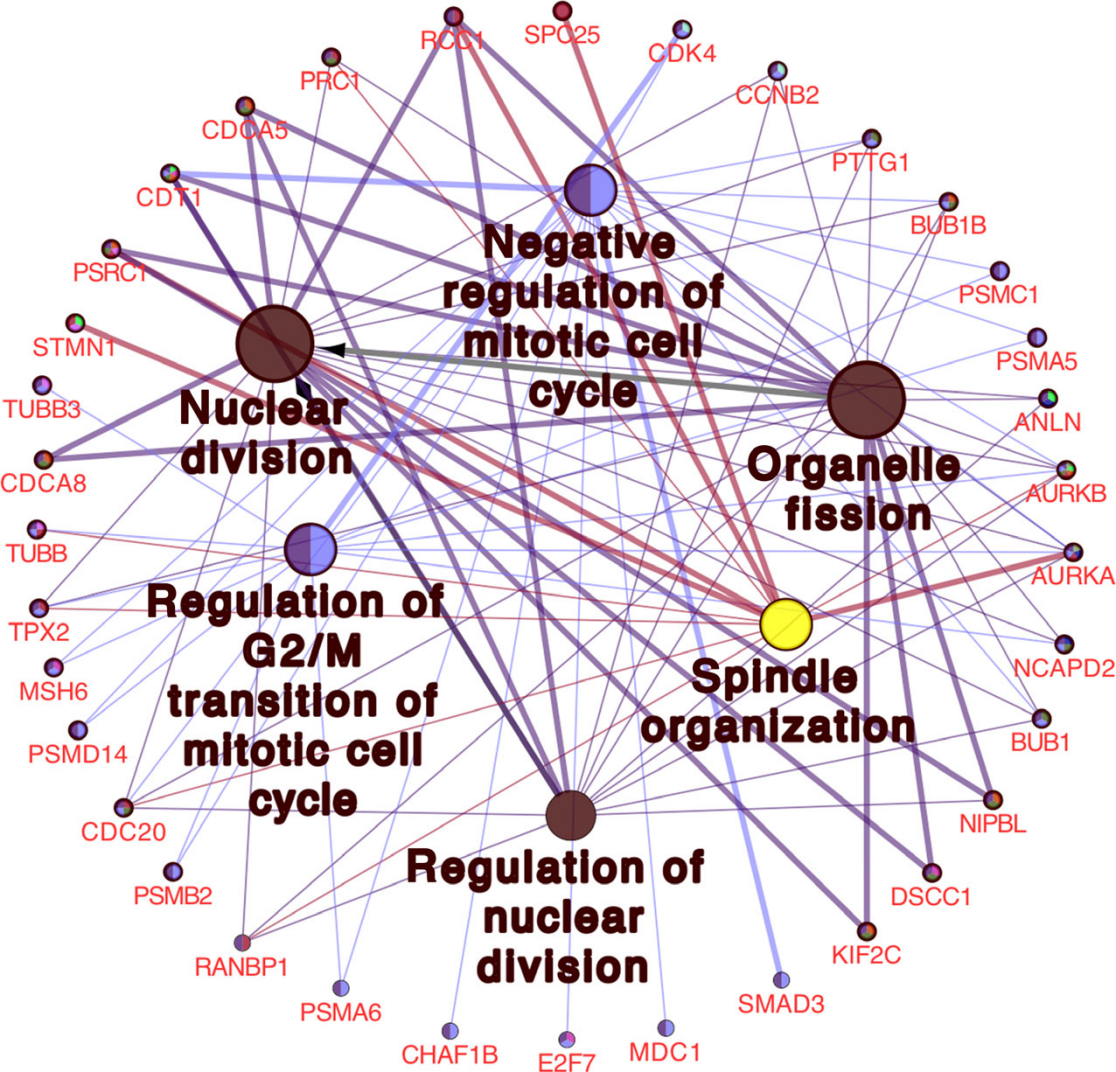
Significant enriched GO terms and involved genes.

**Fig. 7:**
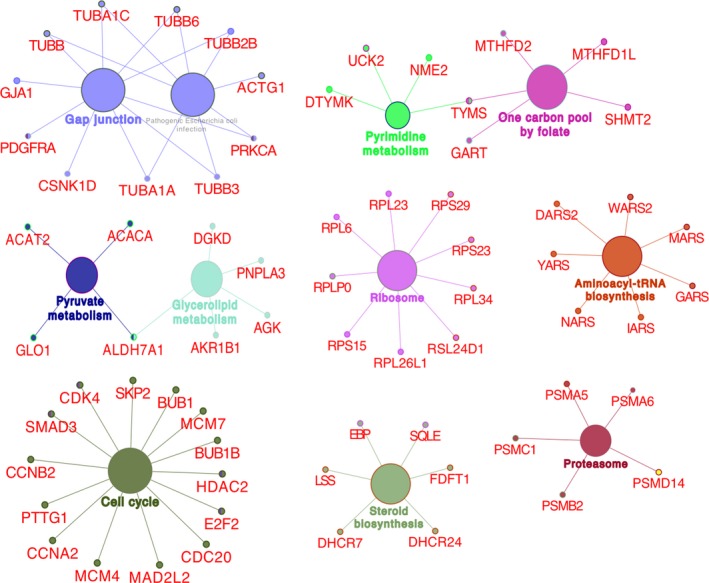
Significant enriched KEGG terms and involved genes.

GO and KEGG enrichment analyses of 514 overlapping genes were conducted by DAVID database, and the enriched terms with *P* < 0.05 and false discovery rate < 0.05 were displayed (Data S2). Eleven terms were significantly clustered by target genes for BPs using GO analysis and shown in Fig. 5A. Besides, KEGG analysis also verified 11 significant pathways. Through further analysis, we found that the significant BP GO terms were tightly associated with cell cycle and mitosis (Fig. 5B). KEGG pathways analysis demonstrated that the target genes of miR‐542‐5p were most related to the regulation of cell cycle. Therefore, we investigated the involved genes in GO (Fig. 6) and KEGG pathways (Fig. 7) to explore the potential mechanisms of miR‐542‐5 in osteosarcoma, which might inspire new insights for targeted therapy.

### PPI network construction and module analysis of target genes

**Fig. 8:**
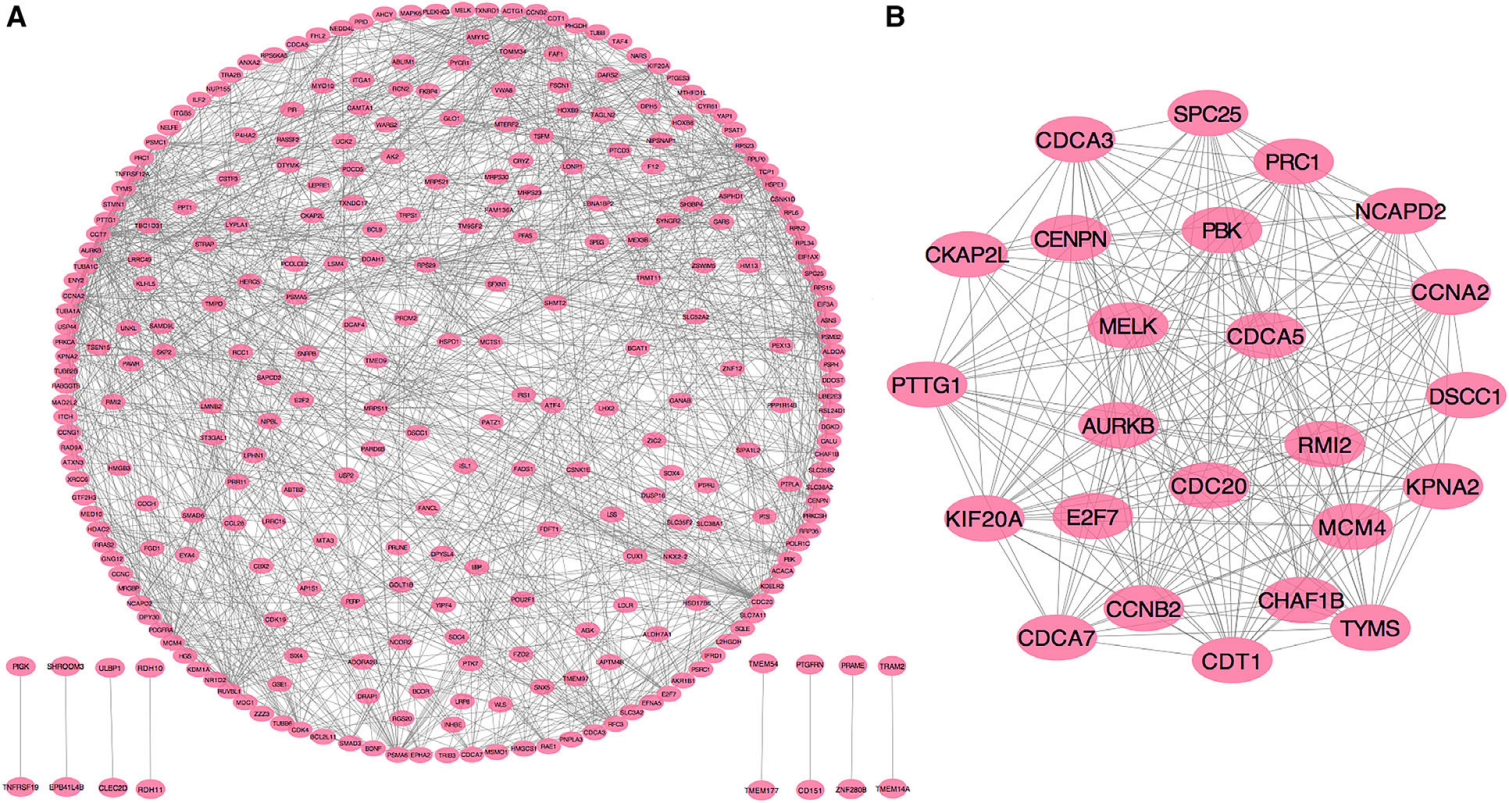
PPI network of possible miR‐542‐5p target genes. (A) The PPI network of differentially expressed genes was constructed using Cytoscape. (B) The most significant module was obtained from the PPI network.

The PPI network of potential targets genes was constructed (Fig. 8A), and the most significant modules were acquired using Cytoscape (Fig. 8B). The most significant modules contained 24 nodes and 230 edges, and each gene was connected with various target genes. Therefore, we identified 11 hub genes (*CDCA5*, *DNMT1*, *UNKL*, *HSPD1*, *KLC1*, *CBX2*, *RPMS17*, *LAMB1*, *SLC3A2*, *PARP12* and *COMMD10*) using Module analysis. Those 11 interactive hub genes may be the key target genes of miR‐542‐5p and involved in regulating signal mechanisms of miR‐542‐5p in osteosarcoma.

### Effect of miR‐542‐5p on cell survival in osteosarcoma cell line U2OS

**Fig. 9:**
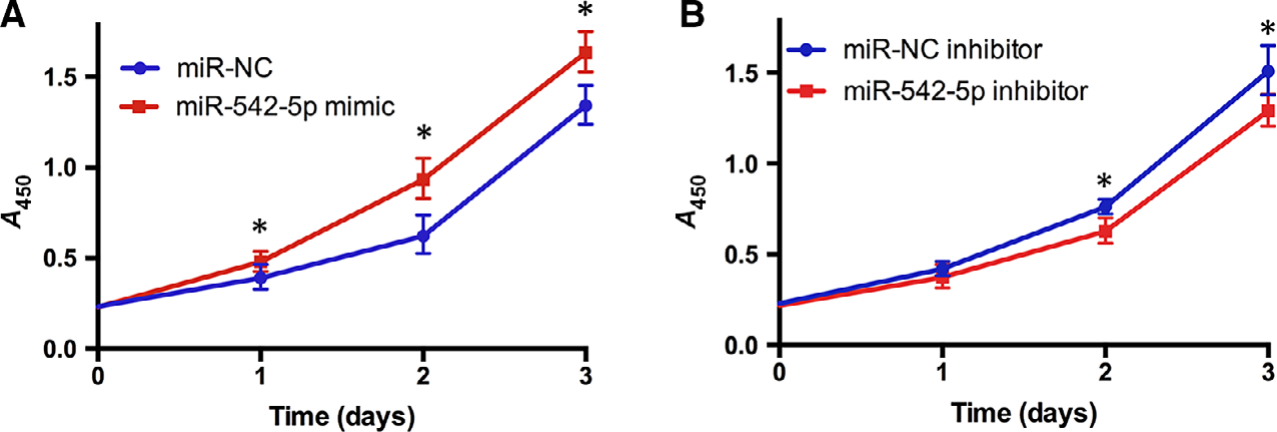
Effects of miR‐542‐5p on the proliferation of osteosarcoma cells. (A) The results of a CCK‐8 assay in U2OS cells transfected with miR‐NC (miR‐Negtive control) or miR‐542‐5p mimic. (B) The results of a CCK‐8 assay in U2OS cells transfected with miR‐NC inhibitor or miR‐542‐5p inhibitor. All results are repeated as the mean ± standard error of the mean and analyzed with Student’s *t*‐test. *P < 0.05.

We examined whether miR‐542‐5p mimic or inhibitor had impacts on osteosarcoma cell line U2OS by CCK‐8 assay. The results indicated that overexpressed miR‐542‐5p promoted osteosarcoma cell proliferation (Fig. 9A). Whereas in the opposite way, down‐regulation of miR‐542‐5p inhibited osteosarcoma cell proliferation (Fig. 9B).

## Discussion

Osteosarcoma is one of the most common malignancies that pose a serious threat to adolescent health [25]. With the continuous improvement of neo‐adjuvant chemotherapy, surgical treatment and comprehensive treatment, the 5‐year survival rate of osteosarcoma with local disease has reached 70%. However, due to the strong invasion ability of osteosarcoma cells and transfer by blood in the early stage, the prognosis of most osteosarcoma patients is not optimistic [2. Therefore, exploring predictive biomarkers and seeking out the molecular mechanisms of osteosarcoma may provide novel perspectives on the diagnosis and treatment of osteosarcoma. Early studies have reported that miRNAs may play important roles in the development and metastasis of osteosarcoma [26].

Currently, more than 700 miRNAs have been identified in humans [27]. miRNAs can act as oncogenes or tumor suppressors through regulating the expression of cancer‐related genes in various tumors [28]. In this study, we intended to find the attractive miRNAs and illuminate the underlying molecular mechanisms in osteosarcoma using a bioinformatics analysis. miR‐542‐5p was selected as the potential candidate in regulating the tumorigenesis of osteosarcoma by overlapping three GEO datasets. The differential expression analysis of osteosarcoma and normal specimens demonstrated that miR‐542‐5p may be a key diagnostic biomarker and potential therapeutic target in osteosarcoma. Several studies have described the functions of miR‐542‐5p in tumors such as lung cancer [29], breast cancer [30] and endometrial carcinosarcoma [31]. However, the role of miR‐542‐5p in osteosarcoma remains unclear, and the mechanisms need further investigation.

We observed that the expression of miR‐542‐5p was significantly increased in osteosarcoma compared with the normal bones. To better understand the molecular role of miR‐542‐5p in osteosarcoma, we investigated GO and KEGG pathway analyses to comprehensively analyze the molecular interactions between the target genes of miR‐542‐5p. According to the GO analysis, the most directly related terms were cell cycle, mitosis, metabolic process and organelle fission, indicating that miR‐542‐5p might impact the development of osteosarcoma by participating in the earlier BPs. Similar to the results of GO, KEGG pathway analysis revealed that cell cycle, gap junction and ribosome were involved in mediating the functions of miR‐542‐5p. Based on those earlier findings, we speculated that miR‐542‐5p participated in regulation of cell cycles. Besides, we constructed the PPI network of the target genes and scheduled the most densely connected region based on topology. *CDCA5*, *DNMT1*, *UNKL*, *HSPD1*, *KLC1*, *CBX2*, *RPMS17*, *LAMB1*, *SLC3A2*, *PARP12* and *COMMD10* were selected as the hub genes, and elucidation of the function of these hub genes might provide new insights into the molecular mechanisms of miR‐542‐5p in osteosarcoma. Furthermore, we conducted experiments by transfected osteosarcoma cell line U2OS with miR‐542‐5p mimics or inhibitors. The results showed that miR‐542‐5p promoted proliferation in osteosarcoma cells, which is consistent with our previous informatics analysis.

## Conclusions

To our knowledge, this was the first study to comprehensively analyze the expression profiles and explore the roles of miRNA in osteosarcoma by integrating bioinformatics analysis. We speculated that miR‐542‐5p may be promising for a new biomarker in the early diagnosis of patients with osteosarcoma based on current research. Further molecular mechanism investigation should be carried out to explore signal pathways associated with miR‐542‐5p in osteosarcoma.

## Conflict of interest

The authors declare no conflict of interest.

## Author contributions

TZ and DF contributed to this article in the aspects of drafting the work and analyzing the data for the work. KY was accountable for the statistics work of the article. BL and ZC revised the paper. ZL and YT made substantial contributions to the conception and design of the work.

## Supporting information


**Data S1.** The list of differentially expressed genes of three miRNA datasets.

**Data S2.** The list of 514 potential target genes of miR‐542‐5p.
